# Probiotic pretreatment improves survival and prevents gut mucosal barrier dysfunction in sepsis

**DOI:** 10.1186/cc14072

**Published:** 2014-12-03

**Authors:** KL Calisto, ACAP Camacho, FC Mittestainer, MCS Mendes, AC Santos, MJA Saad

**Affiliations:** 1 Department of Internal Medicine, Faculty of Medical Sciences, UNICAMP Campinas, Brazil

## Introduction

The gut is the largest immune organ and plays a central role in the promotion of systemic inflammatory responses [1]. Perturbations of intestinal epithelial homeostasis during sepsis include increased proinflammatory cytokine production, increased intestinal permeability and apoptosis [2-6]. Healthy gut is essential to promote host health and prevent organ dysfunction in sepsis. Probiotics seem to keep gut homeostasis through different pathways, such as the modulation of microbial activity, energy regulation, anti-inflammatory cytokine production, gene expression and cell differentiation [7]. Probiotics have been shown an effective treatment in various clinical conditions, although the potential benefits of probiotic treatment in sepsis remain largely undefined. The aim of the present study was to investigate the effect of probiotic treatment on gut dysfunction and inflammatory signaling in septic rats.

## Methods

Sepsis was induced by cecal ligation and puncture (CLP) in Wistar male rats (8 weeks old). They were pretreated with probiotics or vehicle once a day during 7 days before CLP. The chosen probiotic mixture contained 10 × 10^7 ^CFU *Bifidobacterium longum*, 10 × 10^6 ^CFU *Lactobacillus bulgaricus*, 10 × 10^6 ^CFU *Lactobacillus acidophilus*. Colonic tissue and serum samples were collected 24 hours after CLP for ELISA and protein expression analysis by western blotting.

## Results

**Figure 1:**
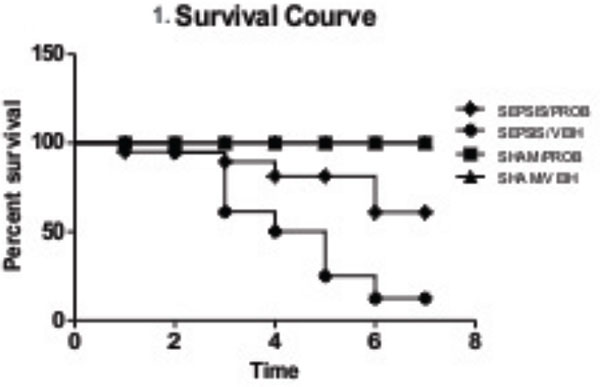


**Figure 2:**
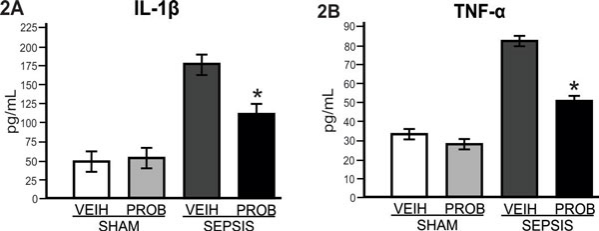


**Figure 3:**
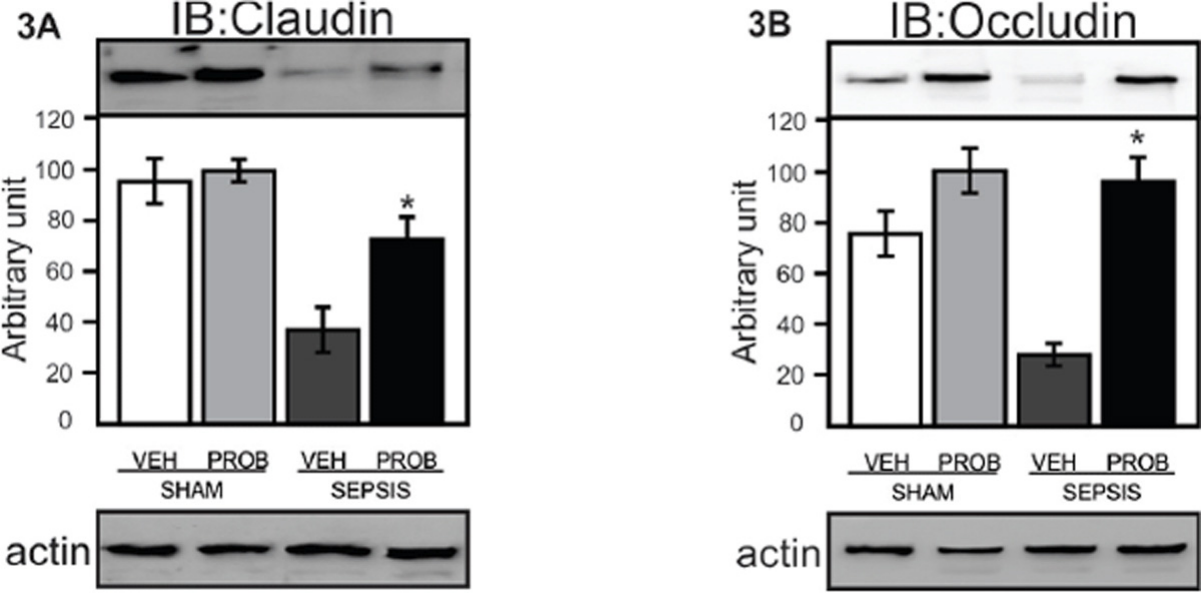


**Figure 4:**
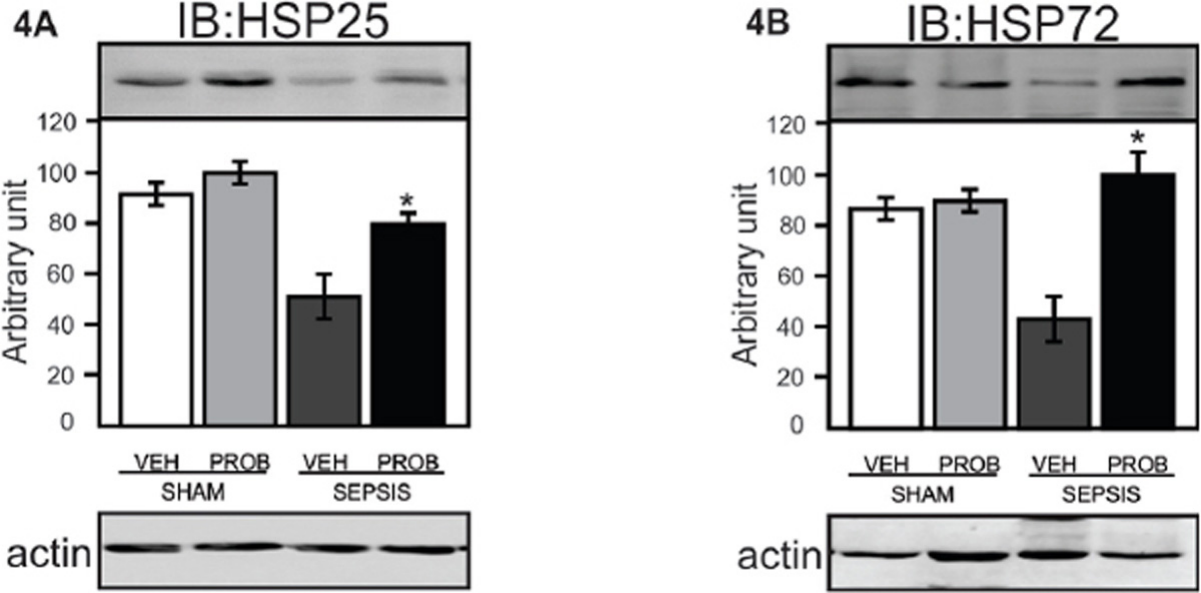


Our data demonstrate that probiotic pretreatment improved survival of septic rats (Figure 1) and this effect is accompanied by a marked decrease of IL-1β and TNFα (Figure 2A,B). Sepsis leads to severe intestinal epithelial damage with a decrease in claudin 2 and occludin protein expression (Figure 3A,B); probiotic pretreatment reversed these alterations in parallel with an increase in Hsp72 and Hsp25 activation (Figure 4A,B). In intestinal epithelial cells, the inducible Hsp have been shown to preserve tight junction and barrier function. The maintenance of epithelial barrier integrity induced by probiotic pretreatment, in parallel with an activation of cytoprotective pathway, may culminate in the restoration of the intestinal epithelial function.

## Conclusion

Our results show that probiotics pretreatment fulfills a dual function at the intestinal mucosa: in addition to preventing intestinal permeability disruption, it also attenuates proinflammatory cytokine release, diminishing the exacerbate host's reaction to infection and offering a novel prophylactic strategy to sepsis.
